# Unmasking an unusual presentation of Hodgkin’s lymphoma masquerading as ocular inflammation: a case report

**DOI:** 10.1186/s13256-024-04613-0

**Published:** 2024-07-04

**Authors:** Anna Zein-El-Din, Rami Abdo, Amina Elbachti, Gabriel Boustani, Dany Salik, Sayeh Pourjavan

**Affiliations:** 1 Department of Ophthalmology, CHIREC Hospital Group, Delta Hospital, Brussels, Belgium; 2 Faculty of Medicine, Université Libre de Bruxelles, Brussels, Belgium; 3 Department of Ophthalmology, Cliniques Universitaires Saint-Luc, UCL, Brussels, Belgium

**Keywords:** Uveitis, Ocular inflammation, Hodgkin’s lymphoma, Case report

## Abstract

**Background:**

Hodgkin’s lymphoma (HL) is an extremely rare cause of ocular inflammation that is usually not considered in the typical workup of uveitis and other eye diseases. A few cases of ocular inflammation were reported previously showcasing HL with absence of typical symptoms of HL at presentation. Acknowledging the potential ocular inflammation associated with HL can prompt ophthalmologists to broaden their diagnostic approach and collaborate with internal medicine departments to investigate this rare yet significant etiology.

**Case presentation:**

A 17-year-old Caucasian woman presenting unilateral panuveitis was later diagnosed with HL. The ocular findings were non-necrotizing scleritis, anterior uveitis, vitritis, white/yellowish chorioretinal lesions, papillitis and vasculitis. A left supra-clavicular lymph node biopsy confirmed the diagnosis of nodular sclerosing Hodgkin’s lymphoma stage IIB. Other causes of uveitis were excluded. Chemotherapy led to remission of the disease and the ocular lesions became quiescent with persistent pigmented chorioretinal scars.

**Conclusions:**

Hodgkin's lymphoma should be considered in the differential diagnosis of diseases that can occasionally be revealed by unilateral ocular inflammation. A comprehensive, multidisciplinary approach is key to properly assessing such cases.

## Introduction

Hodgkin’s lymphoma (HL) is a malignant neoplasm arising mainly from B cells characterized by the spread of tumor cells along the lymphoid system, giving localized nodal disease with often indolent clinical presentation. Symptoms of HL encompass a variety of clinical presentations ranging from indolent lymph nodes and enlarged spleen to the presence of B symptoms including fever, night sweats, and/or unexplained loss of body weight > 10% within the preceding 6 months. Nonetheless, intraocular manifestations are extremely rarely associated with this disease. HL predominantly affects young individuals regardless of gender, with a heightened risk among immunosuppressed patients.

This case reports a young 17-year-old patient with an atypical unilateral inflammatory ocular pathology, later diagnosed as potentially linked to Hodgkin’s lymphoma. The diagnosis became feasible following an extensive investigation aimed at uncovering the root cause of the patient's persistent inflammatory symptoms.

## Case report

A 17-year-old Caucasian female presented to our ophthalmic emergency department reporting right eye redness, pain, and blurred vision along with intermittent temporal photopsia with onset 5 days before consultation. General symptoms were vague encompassing nausea, headaches, left shoulder pain and long-standing night sweats. The patient’s history was significant for right eye conjunctivitis, conjunctival hyperhemia and papillary edema since 2017 but best corrected visual acuity (BCVA) (using decimal VA notation) remained 1.0 in both eyes. No underlying disease was diagnosed despite thorough investigations including blood testing, magnetic resonance imaging (MRI) and neurological examinations; these previous episodes resolved after brief local corticosteroid treatments.

**Fig. 1:**
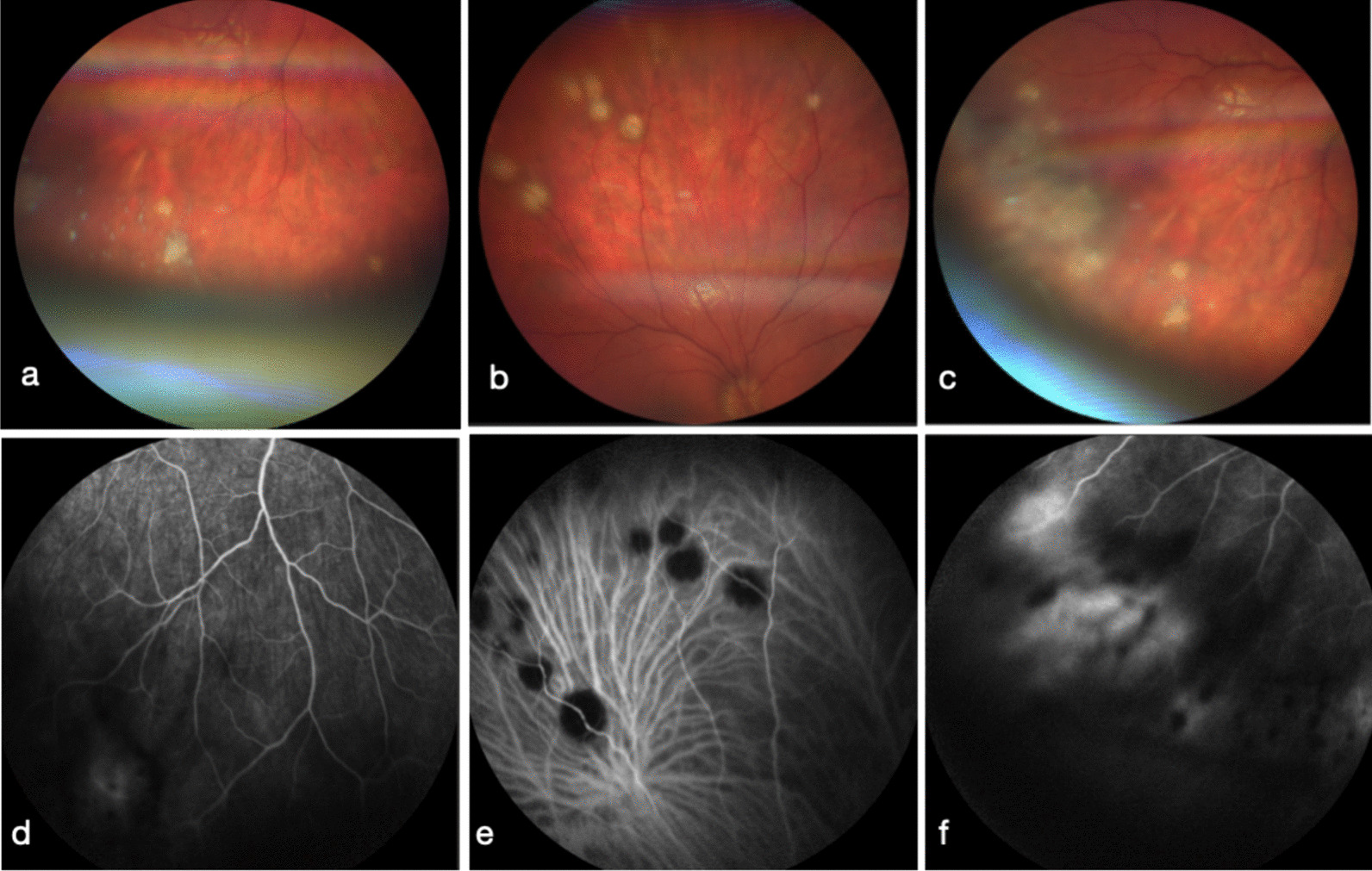
Active unilateral right eye (RE) panuveitis at presentation. **a**–**c** Color photographs of the right posterior pole, superior and inferior temporal white/yellowish round chorioretinal lesions. **d**–**f** Fluoroangiography (**d**, **f**) and indocyanine green angiography (**e**) showing venous vasculitis, with hyperfluorescent chorioretinal lesions and peripheral hypofluorescent spots

**Fig. 2:**
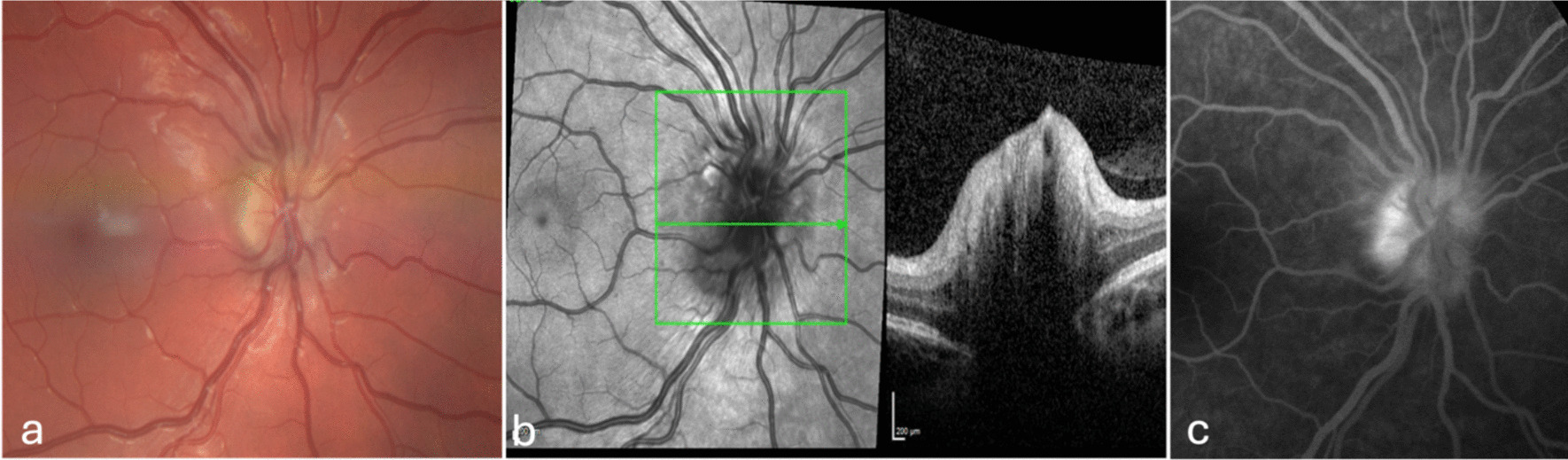
Color fundus (**a**) and optical coherence tomography image (**b**) showing RE papillary edema and FA (**c**) showing a hot disc aspect at presentation

**Fig. 3:**
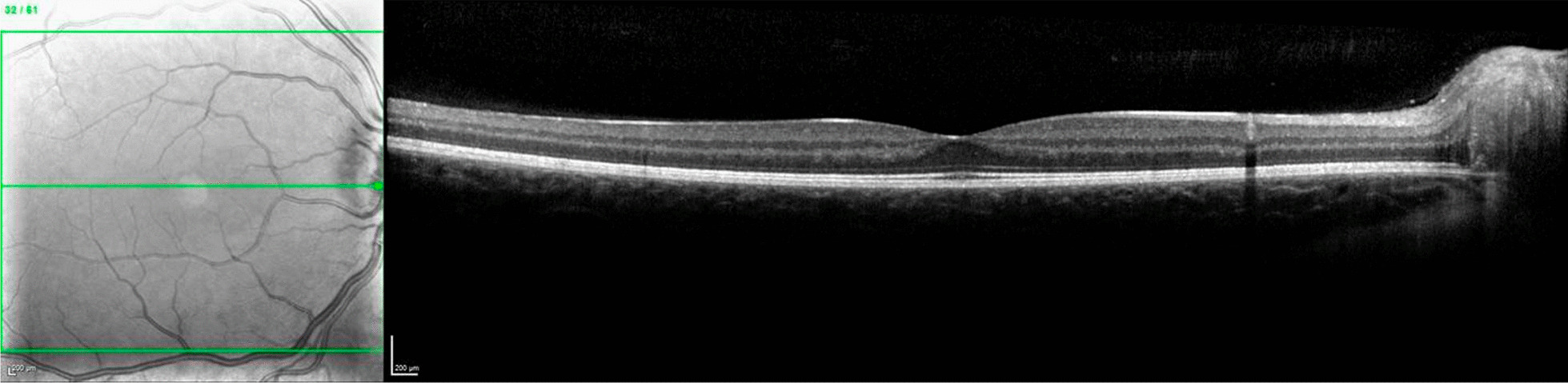
Optical coherence tomography showing normal aspect of RE macular retinal layers at presentation

On presentation, the patient’s BCVA was 1.0 in both eyes. Initial slit lamp examination revealed right eye non-necrotizing scleritis, endothelial haze and 1 + cells in the anterior chamber. Upon fundus examination, right eye vitreous turbidity, inferior vitreous condensations and vitreous snowballs in the inferior periphery were visualized (Fig. 1a). Papillary edema (Fig. 2) was also present as well as peripheral white/yellowish lesions in the superior and inferior retinal peripheries (Fig. 1b, c). Fluoroangiography (FA) and indocyanine green angiography (ICGA) showed early arterial filling of central choriocapillaris, peripheral vasculitis, a hot disc papillitis appearance and peripheral hypofluorescent spots (Fig. 1d–f). Spectral Domain OCT (SD-OCT) did not show any alterations in retinal layers, retinal pigment epithelium (RPE) or the vitreoretinal interface (Fig. 3).

The left eye slit lamp examination, fundus examination, FA, ICGA and SD-OCT were strictly normal.

The patient was initially treated with local hourly Prednisolone (1%), tropicamide (0.5%) twice daily and oral non-steroidal anti-inflammatory drugs (NSAIDs) (Ibuprofen EG 600 mg twice daily) for pain relief.

Subsequent weekly follow-ups were significant for increased flashes and worsening of RE pain and BCVA of 0.8, 0.5 + cells in the anterior chamber, persistent scleritis and worsening of vitreous condensations, progression of the papillary edema and emergence of 2 new peripheral retinal white/yellowish lesions.

Collaboration with the internal medicine department was underway to investigate the underlying causes of the panuveitis and recurrent scleritis presented by the patient. At time of presentation, the patient’s physical examination showed normal lymph nodes as well as normal neurological, cardiac, and pulmonary examinations.

In light of the papillary changes, a brain MRI was conducted, revealing no abnormalities.

A complete workup ruling out the following diagnosis came back negative: infectious causes such as Toxoplasmosis, Whipple's disease, Syphilis, Lyme disease, Cat scratch disease, Herpes simplex virus, Varicella-zoster virus, Cytomegalovirus as well as systemic disease: sarcoidosis, Behçet’s disease and systemic lupus erythematosus (SLE). IGRA testing for tuberculosis came back positive but with negative Quantiferon.

Treatment with oral corticosteroids (48 mg/day) was proposed in collaboration with the internal medicine team.

**Fig. 4:**
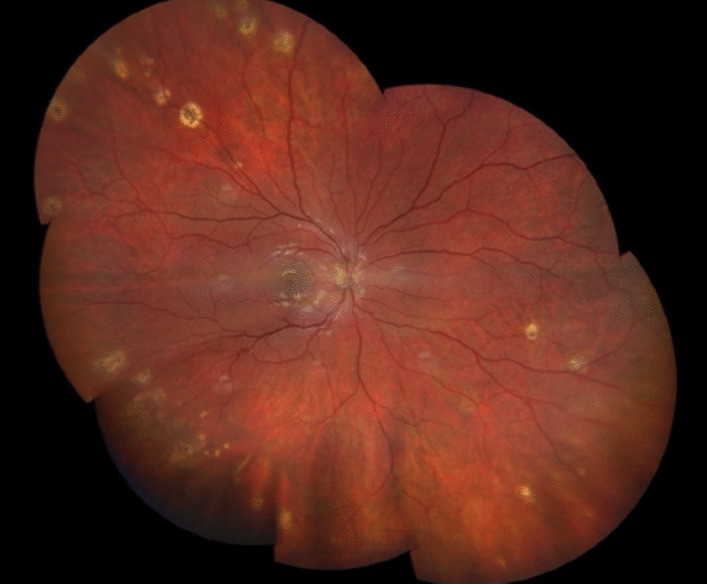
Fundus imaging at the remission stage shows pigmented chorioretinal lesions and peripapillary fibrosis

At follow-up, BCVA and pain gradually improved to 0.9 and then stabilized at 1.0 with persistent photopsia. The vitreous reaction as well as retinal lesions aspects were showing signs of early regression (Fig. 4).

Considering the clinical improvement, oral corticosteroids were progressively tapered with a final dose of 8 mg/day. Locally, topical corticoids were maintained at a frequency of twice per day.

Further workup consisted of a positron emission tomography-computed tomography (PET-CT) scan for exclusion of extra-pulmonary tuberculosis in light of positive IGRA which revealed hypermetabolic left supra-clavicular and mediastinal bilateral lymph nodes leading to the differential diagnosis of lymph node tuberculosis, lymphoma, sarcoidosis, IgG4 related disease or Castelman’s disease.

A bronchioalveolar lavage was carried out with subsequent negative results. A lymph node biopsy came back positive for nodular sclerosing Hodgkin’s lymphoma stage IIB.

Subsequently, the patient underwent 6 cycles of ABVD (doxorubicin, bleomycin, vinblastine, dacarbazine) chemotherapy. With the approval of the oncology team, topical and systemic corticosteroids were maintained at the same dosages, and the patient was followed biweekly, showing adequate tolerability to chemotherapy and good adherence to local treatment, with BCVA staying stable, absence of pain but persistence of intermittent temporal right eye photopsia that may be linked to the presence of chorioretinal scarring.

## Discussion

This case report showcases yet another case of panuveitis leading to the diagnosis of Hodgkin’s lymphoma, which is a type of lymphoma in which cancer originates from a specific type of white blood cells, lymphocytes, characterized by the presence of typical multinucleated Reed-Sternberg cells and mononuclear Hodgkin cells (both accounting for only 10% of the cells constituting these tumors) in a highly inflammatory background formed by B cells, T cells, histiocytes, neutrophils, eosinophils, mast cells and plasma cells [1]. HL accounts for 15–20% of lymphomas in Western countries and has a bimodal distribution with two peaks occurring in early (15–35 years) and late (after age 55) adulthood [2]. Young patients, being mainly affected by the disease are good responders to chemotherapy (80–90%), one of the main treatments employed nowadays, next to radiation and targeted therapies. HL can be classified as nodular lymphocyte predominant or classic HL (nodular sclerosing, mixed cellularity HL, lymphocyte rich HL and lymphocyte depleted subtype). The disease mainly affects peripheral lymph nodes, mediastinal lymph nodes that may advance to infiltrate the spleen, liver or extranodal locations but with a constant nodal concomitant disease [2].

Symptoms associated with HL include a variety of clinical presentations ranging from indolent lymph nodes and enlarged spleen to the presence of B symptoms including fever, night sweats, and/or unexplained loss of body weight > 10% within the preceding 6 months [3]. Diagnosis of the disease is classically made by histopathological evaluation of lymph nodes or punch biopsies of the affected organs [4]. As for the staging, apart from histopathological evaluation, detailed history, clinical examination as well as imaging modalities (contrast-enhanced computed tomography (ceCT) and 18FDG-PET/CT) are necessary [5].

Based on the various publications on the few similar cases and the discussion within our medical team, we considered two main hypotheses regarding the chorioretinal lesions in our patient: either the presence of lymphomatous cells in the choroid or a paraneoplastic manifestation. Hematogenous spread of malignant cells to the choroid is not a known phenomenon in HL. Therefore, we considered that the observed condition was more likely an unusual paraneoplastic syndrome. Notably, while uncommon, Cancer Associated Retinopathies (CAR) stand out as the most prevalent type of paraneoplastic autoimmune retinopathies (AIR).

This syndrome’s mechanisms stem from an interaction between antibodies targeting tumor antigens and retinal proteins. Patients with CAR express various antibodies [6], notably anti-recoverin (23 kD) targeting photoreceptor cells, linked primarily to small-cell lung and gynecologic cancers [7]. Melanoma associated retinopathy (MAR), encountered in uncontrolled melanomas, and bilateral diffuse uveal melanocytic proliferation (BDUMP), are also forms of paraneoplastic AIR [8]. While our case shares some similarities with CAR, MAR, or BDUMP, it presents distinctive features. Unlike typical cases of these disorders, our patient's condition does not lead to a progressive bilateral irreversible decline in vision with narrowed visual fields. Furthermore, manifestations typically include vasculitis, mild vitritis, late-stage salt-and-pepper retinal aspects, and optic atrophy, which were not observed in our case.

Within the context of Hodgkin's lymphoma, there have been rare occurrences of retinopathies. These manifestations typically present bilaterally and may encompass anterior uveitis, occasionally described as granulomatous. Additionally, reported signs include white chorioretinal lesions, cystoid macular edema, papillitis, moderate vitritis, and vasculitis [9–13].

In other occurrences, bilateral optic disc swelling, periphlebitis, neuroretinitis and focal chorioretinitis were observed [10, 14–16].

In one isolated case, granulomatous inflammation devoid of tumoral cells was observed following vitrectomy [17].

Some reports of more anterior involvement such as conjunctivitis [18], corneal inflammation [19], episcleritis and slceritis [20] related to HL were also previously made.

A serum antibody reacting to a 65 kD retinal protein was detected in a case of lymphoma-associated retinopathy [7]. This bolstered the hypothesis that a paraneoplastic mechanism, associated with an immunological cross-reaction, is more likely than intraocular proliferation of lymphomatous cells.

In the majority of cases, achieving remission of Hodgkin's lymphoma led to the disappearance of ocular lesions. However, the visual prognosis varied considerably, primarily depending on the extent of initial involvement [9]. Therefore, a regular follow-up and adaptation of systemic treatment of HL along with local ophthalmic treatment may facilitate complete regression of symptoms, better preservation of vision and may prevent permanent damage to the affect part of the eye [21]. Likewise, in our patient, collaboration with internal medicine and oncology teams led to a prompt examination and a timely diagnosis that allowed halting of the progression of the panuveitis most probably being a paraneoplastic symptom of HL. While chorioretinal scarring was still present in our patient, her BCVA and symptoms such as pain were halted due to local and systemic anti-inflammatory treatment as well as general treatment of HL by chemotherapy sessions. A consistent ophthalmological follow-up would be of high importance to conclude whether regression of HL might cease ocular inflammation and its associated symptoms.

Despite our meticulous approach to this case, which involved thorough investigation of all notable differential diagnoses and close collaboration with internal medicine and oncology teams, some limitations were present, prompting reflection. Clinically, we did not obtain objective evidence of HL in the eye through methods such as vitreous tap or biopsy. However, we opted for a non-invasive approach as invasive procedures were deemed unnecessary from a clinical standpoint. Additionally, further longitudinal assessment of retinal lesions and continued patient follow-up are essential to observe the long-term effects of chemotherapy and local treatments on the disease. It's essential to acknowledge that our observations and discussions are limited by the timeframe after the patient's consultation.

## Conclusion

This case emphasizes the importance of a meticulous investigation and a multidisciplinary approach to ocular presentations leading to consideration of both common and rare entities. In our patient, panuveitis and recurring scleritis led to an extensive investigation ultimately revealing an underlying scleronodular Hodgkin’s lymphoma, a rarely described cause of ocular inflammation. Timely collaboration with internal medicine and oncology departments facilitated successful management and halted the progression of ocular inflammation, highlighting the importance of considering HL as a rare systemic etiology capable of manifesting as an ocular inflammation at patient’s presentation.

## Data Availability

On request.
